# Transposons passively and actively contribute to evolution of the two-speed genome of a fungal pathogen

**DOI:** 10.1101/gr.204974.116

**Published:** 2016-08

**Authors:** Luigi Faino, Michael F. Seidl, Xiaoqian Shi-Kunne, Marc Pauper, Grardy C.M. van den Berg, Alexander H.J. Wittenberg, Bart P.H.J. Thomma

**Affiliations:** 1 Laboratory of Phytopathology, Wageningen University, 6708 PB Wageningen, The Netherlands;; 2 Keygene N.V., 6708 PW Wageningen, The Netherlands

## Abstract

Genomic plasticity enables adaptation to changing environments, which is especially relevant for pathogens that engage in “arms races” with their hosts. In many pathogens, genes mediating virulence cluster in highly variable, transposon-rich, physically distinct genomic compartments. However, understanding of the evolution of these compartments, and the role of transposons therein, remains limited. Here, we show that transposons are the major driving force for adaptive genome evolution in the fungal plant pathogen *Verticillium dahliae*. We show that highly variable lineage-specific (LS) regions evolved by genomic rearrangements that are mediated by erroneous double-strand repair, often utilizing transposons. We furthermore show that recent genetic duplications are enhanced in LS regions, against an older episode of duplication events. Finally, LS regions are enriched in active transposons, which contribute to local genome plasticity. Thus, we provide evidence for genome shaping by transposons, both in an active and passive manner, which impacts the evolution of pathogen virulence.

Genomic plasticity enables organisms to adapt to environmental changes and occupy novel niches. Although such adaptation occurs in any organism, this is particularly relevant for pathogens that engage in coevolutionary “arms races” with their hosts (Raffaele and Kamoun 2012; Seidl and Thomma 2014; Dong et al. 2015). In these interactions, hosts utilize their surveillance system to detect invaders and mount appropriate defenses, involving detection of invasion patterns by immune receptors, whereas pathogens secrete so-called effector molecules to support host colonization and counteract immune responses (Rovenich et al. 2014; Cook et al. 2015). This tight interaction exerts strong selection pressure on both partners and incites rapid genomic diversification (McDonald and Linde 2002).

Although sexual reproduction drives genotypic diversity, not all eukaryotes regularly reproduce sexually, including many asexual fungal phyla (McDonald and Linde 2002; Heitman et al. 2007; Flot et al. 2013). However, in such asexual organisms adaptive genome evolution also occurs, mediated by various mechanisms ranging from single-nucleotide polymorphisms to large-scale structural variations that can affect chromosomal shape, organization, and gene content (Seidl and Thomma 2014). *Verticillium dahliae* is a soil-borne fungal pathogen that infects susceptible hosts through their roots and colonizes the water-conducting xylem vessels, leading to vascular wilt disease (Fradin and Thomma 2006). Despite its presumed asexual nature, *V. dahliae* is a highly successful pathogen that causes disease on hundreds of plant hosts (Fradin and Thomma 2006; Klosterman et al. 2009; Inderbitzin and Subbarao 2014). Using comparative genomics, we recently identified genomic rearrangements in *V. dahliae* that lead to extensive chromosomal length polymorphisms (de Jonge et al. 2013). Moreover, the genomes of *V. dahliae* strains were found to contain highly dynamic, repeat-rich, lineage-specific (LS) regions (Klosterman et al. 2011; de Jonge et al. 2013). Intriguingly, these LS regions are enriched for *in planta*-induced effector genes that contribute to fungal virulence (de Jonge et al. 2013). Similarly, many filamentous pathogens are considered to have evolved so-called “two-speed” genomes with gene-rich, repeat-poor genomic compartments that contain core genes that mediate general physiology and evolve slowly, whereas plastic, gene-poor and repeat-rich compartments are enriched in effector genes that mediate virulence in interactions with host plants and evolve relatively quickly (Raffaele and Kamoun 2012).

Plastic, fast-evolving genomic compartments in plant and animal pathogen genomes concern particular regions that are either embedded within the core chromosomes or reside on conditionally dispensable chromosomes (Thon et al. 2006; Fedorova et al. 2008; Haas et al. 2009; Ma et al. 2010; Goodwin et al. 2011; Klosterman et al. 2011; Rouxel et al. 2011; de Jonge et al. 2013). Irrespective of the type of organization of the two-speed genome, the fast-evolving compartment is generally enriched for transposable elements (TEs) that are thought to actively promote genomic changes by causing DNA breaks during excision or by acting as a substrate for rearrangement (Seidl and Thomma 2014). Nevertheless, the exact role of TEs in the evolution of effector genes currently remains unknown (Dong et al. 2015). We recently established gapless *V. dahliae* whole-genome assemblies using a combination of long-read sequencing and optical mapping (Faino et al. 2015; Thomma et al. 2015). Here, we exploit these novel assemblies for in-depth investigations into the molecular mechanisms responsible for genomic variability, which is instrumental for adaptive genome evolution in *V. dahliae*.

## Results

### Genomic rearrangements in *Verticillium dahliae* are associated with sequence similarity

**Figure 1.**
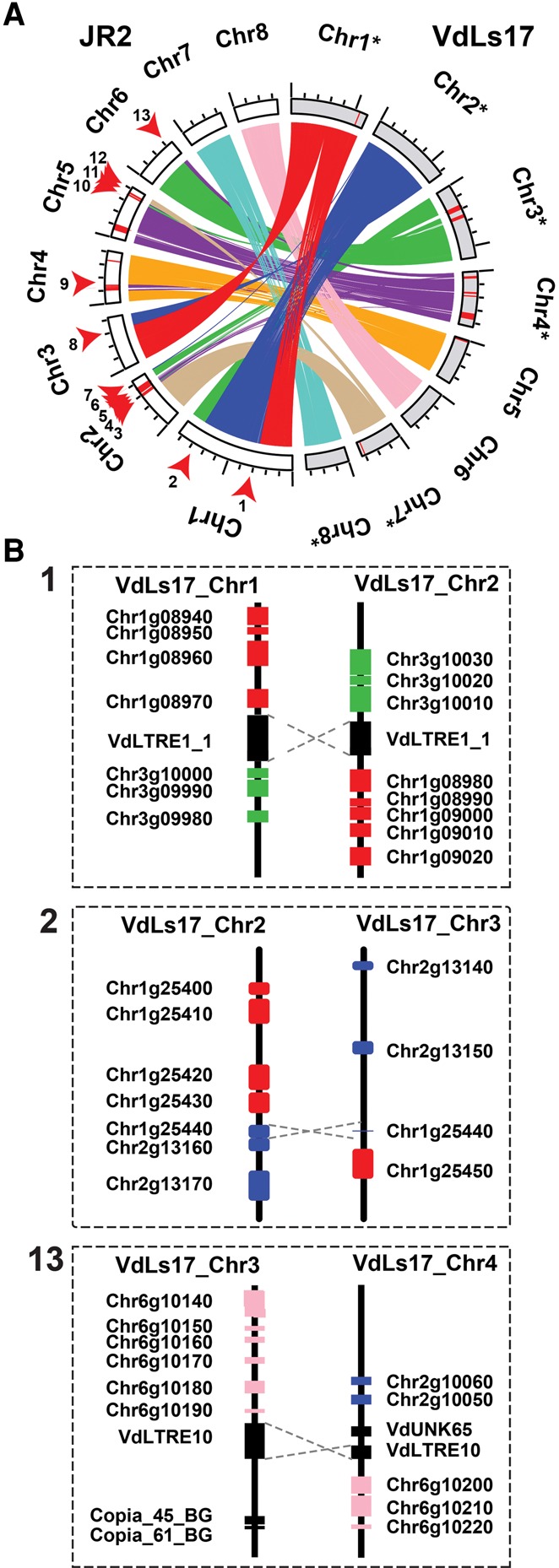
Extensive rearrangements in *Verticillium dahliae* genomes are mediated by repetitive elements. (*A*) Syntenic regions, indicated by ribbons, between chromosomes of the two highly similar *V. dahliae* strains JR2 (chromosomes displayed in white) and VdLs17 (chromosomes displayed in gray) reveal multiple synteny breakpoints caused by inter-chromosomal rearrangements, highlighted by red arrows for the JR2 genome. Red bars on the chromosomes indicate lineage-specific genomic regions (LS) that lack synteny in the other strain. To facilitate visibility, some chromosomes of *V. dahliae* strain VdLs17 have been reversed and complemented (indicated by asterisks). (*B*) Detailed view of the genomic regions surrounding selected synteny breakpoints. Rearrangements over short homologous regions such as repetitive elements (black boxes) or genes (colored boxes) resulted in inter-chromosomal rearrangements (translocations). *V. dahliae* strain VdLs17 genes were inferred by mapping of the *V. dahliae* strain JR2 genes to the genome assembly of *V. dahliae* strain VdLs17. Dashed gray lines indicate rearrangement sites. The numbers correspond to rearrangement numbers in *A* and Table 1.

Whole-genome alignments between *V. dahliae* strains JR2 and VdLs17 revealed 24 synteny disruptions (Fig. 1A; Supplemental Fig. S1). To further increase their resolution, we aligned long (average ∼9 kb) sequencing reads derived from strain VdLs17 (Faino et al. 2015) to the strain JR2 assembly, and reads of which the two sides aligned to two distinct genomic locations were used to determine breakpoints at high-resolution (Supplemental Fig. S2). After manual refinement, this procedure yielded 19 large-scale chromosomal rearrangements; 13 inter-chromosomal translocations, and six intra-chromosomal inversions (Fig. 1; Table 1). Although seven rearrangements were confined to regions smaller than 100 bp, the remaining 12 concern larger genomic regions that could not be further refined due to the presence of strain-specific or repeat-rich regions (Fig. 1A).

**Table 1.**
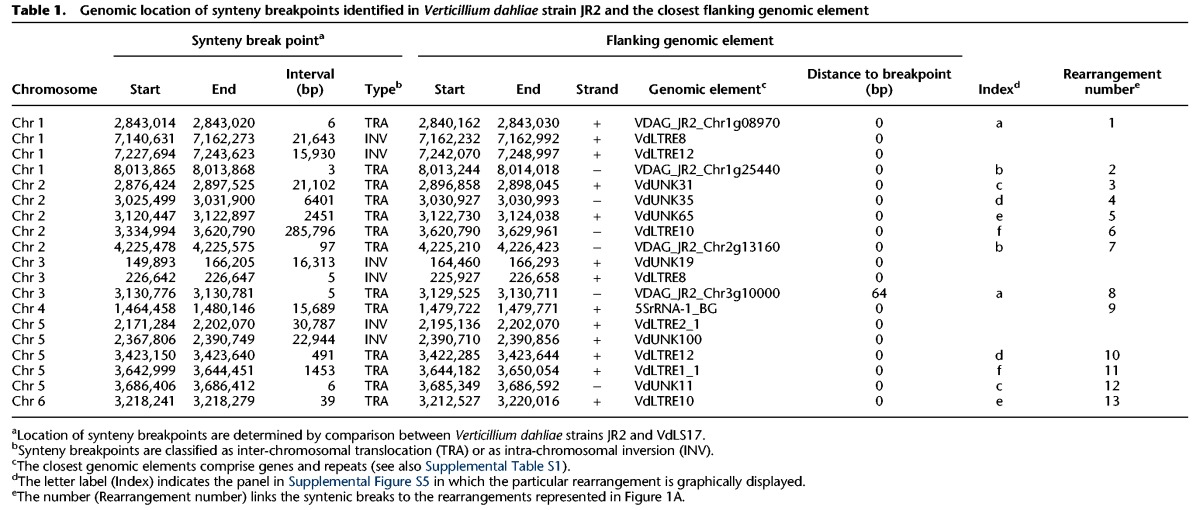
Genomic location of synteny breakpoints identified in *Verticillium dahliae* strain JR2 and the closest flanking genomic element

We subsequently assessed the occurrence of the 13 inter-chromosomal rearrangements in nine other *V. dahliae* strains by querying paired-end reads derived from these genomes for pairs of discordantly mapped reads (i.e., both reads fail to map at the expected distance or location) when mapped onto the strain JR2 assembly, revealing distinct rearrangement patterns (Supplemental Fig. S3). Although some synteny breakpoints identified in *V. dahliae* strain JR2 are either specific to *V. dahliae* strain VdLs17 (Chr 1: 2,843,014–2,843,020) (Supplemental Fig. S4A) or common to all other *V. dahliae* strains (Chr 1: 8,013,865–8,013,868) (Supplemental Fig. S4B), some are observed in only a subset of *V. dahliae* strains (Chr 3: 3,130,776–3,130,781) (Supplemental Fig. S4C).

*V. dahliae* strains JR2 and VdLs17 are highly similar with only 8622 SNPs difference (0.024% nucleotide divergence). When focusing on the regions (10 kb) upstream of and downstream from the 19 identified synteny breakpoints, we observe ∼200 SNPs (∼0.05% divergence), indicating a moderate increase in proximity of the breakpoints. Similarly, when considering all *V. dahliae* strains, we observe a genome-wide average of 4.1 SNPs/kb; whereas around breakpoints (10 kb upstream and downstream), 6.3 SNPs/kb are found.

Genomic rearrangements are generally caused by double-strand DNA breakages followed by unfaithful repair by mechanisms that utilize homologous sequences to repair such breaks (Hedges and Deininger 2007; Krejci et al. 2012; Seidl and Thomma 2014). Repetitive genomic elements, such as transposable elements (TEs), may give rise to genomic rearrangements by providing an ectopic substrate that interferes with faithful repair of the original break. Notably, of 19 chromosomal rearrangements identified in *V. dahliae* strain JR2, 15 colocalize with a repetitive element (Table 1; Supplemental Table S1). Of the 13 inter-chromosomal rearrangements, 12 could be reconstructed in detail (Fig. 1A; Table 1; Supplemental Figs. S5, S6). Eight of them occur over highly similar TEs in both strains, although they belong to different TE families (Fig. 1B; Table 1; Supplemental Figs S5, S6). At two of the breakpoints, a TE occurred in strain VdLs17 that was absent at those breakpoints in the JR2 strain. For the final two breakpoints, no association to a TE was found in either strain, but extended sequence similarity surrounding the rearrangement site was identified (Fig. 1B; Supplemental Fig. S6). Therefore, we conclude that not necessarily TEs or their activity, but rather stretches of sequence similarity, are associated with ectopic chromosomal rearrangements in *V. dahliae*, likely mediating unfaithful homology-based DNA repair. Since TEs are more abundant compared to other (types of) sequences, these are more likely to become substrates for double-strand repair pathways.

### Lineage-specific genomic regions in *Verticillium dahliae* evolved by segmental genomic duplications

Whole-genome alignments between *V. dahliae* strains JR2 and VdLs17 revealed four large (>10 kb) repeat-rich genomic regions lacking synteny between the strains (Supplemental Table S2). Mapping of reads from nine additional strains (de Jonge et al. 2012) onto the JR2 and VdLs17 assemblies revealed absence of read coverage primarily at their respective LS regions (Supplemental Fig. S7). Notably, several inter-chromosomal rearrangements colocalize with these LS regions (Fig. 1A), suggesting that genomic rearrangements contributed to the formation of LS regions.

**Figure 2.**
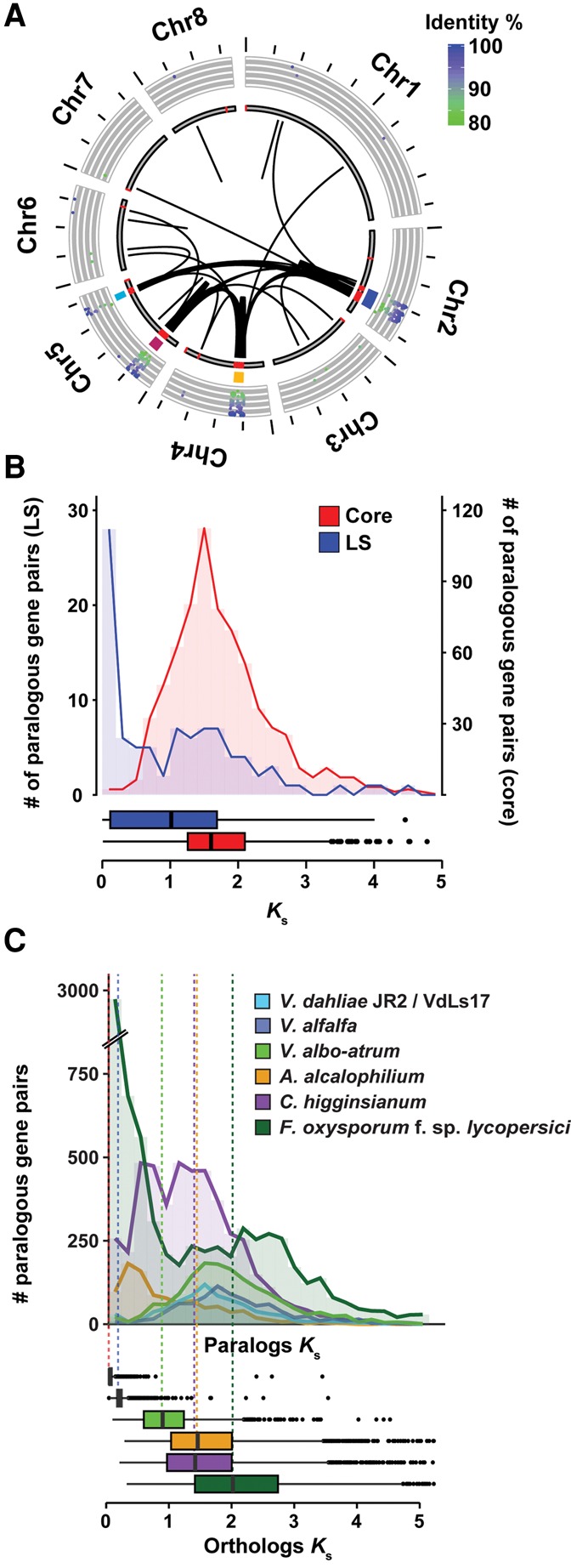
Whole-genome alignments of *Verticillium dahliae* strain JR2 reveals two duplication events. (*A*) Circos diagram illustrating sequence alignments within *V. dahliae* strain JR2. Black lines indicate genomic regions with sequence similarity. The *inner* circle shows LS regions (red lines), the *middle* circle indicates clusters of LS regions, and the *outer* circle shows the identity between pairs of secondary alignments. Each cluster of LS region is color coded: LS1 in blue, LS2 in yellow, LS3 in magenta, and LS4 in light blue (see Supplemental Table S2). (*B*) *K*_s_ distribution of paralogs of which both genes are located in the core genome (red) or at least one paralog is located in an LS region (blue). (*C*) Duplication events are estimated by calculating the *K*_s_ value for paralogous gene pairs and displayed in the line graph. Speciation events are estimated by calculating the *K*_s_ value for orthologous gene pairs based on genes from *V. dahliae* strains JR2 and their respective orthologs in the other genomes and displayed in the box plot. Distributions and median divergence times between 1:1:1 orthologous pairs, displayed by box plots, were used to estimate relative speciation events.

Although duplicated genes have previously been observed in LS regions of *V. dahliae* (Klosterman et al. 2011), the extent of such duplications and their role in the evolution of LS regions remains unknown. To determine the extent of segmental duplications in LS regions, we used two approaches. First, we performed whole-genome nucleotide alignments of *V. dahliae* strain JR2 to itself to identify highly similar (>80% identity), large-scale duplication events, showing that the vast majority of highly similar large-scale duplications occurs within LS regions (Fig. 2A; Supplemental Table S3). Next, we performed homology detection between protein-coding genes in *V. dahliae* strain JR2, establishing a set of approximately 1000 paralogous sequences (Supplemental Fig. S8). Notably, 40% of the 418 genes located in LS regions have a paralog, which is a 4.5× enrichment when compared to the core genome, in which only 7% of the ∼11,000 genes has a paralog (hypergeometric test; *P* = 1.31 × 10^−69^). Therefore, duplications of genomic material are important for the constitution of LS regions in *V. dahliae*.

The high level of similarity between sequences located at LS regions (Fig. 2A; Supplemental Table S3) suggests that a significant proportion of duplications occurred rather recently. To firmly establish when these duplications occurred during the evolution of *V. dahliae*, we used the rate of synonymous substitutions per synonymous site (*K*_s_) between paralogous gene pairs as a proxy for time since these sequences diverged (Fig. 2B). Although the *K*_s_ distribution of paralogous pairs located in the core genome displays a single peak, indicating a single and distinct period in which the majority of these duplications occurred, the distribution of paralogous pairs in which at least one gene is located in the LS regions displays two distinct peaks (Fig. 2B). Notably, the older of the two peaks coincides with the peak observed for the core paralogs, indicating that the expansion of core genes and a subset of genes in LS regions occurred in the same period. The additional peak points toward duplications that occurred more recently. To place these periods in relation to speciation events, we estimated *K*_s_ distributions for orthologous gene pairs between *V. dahliae* strain JR2 and a number of closely related fungi from the taxonomic class of Hypocreomycetidae (Fig. 2C). Within this group of close relatives, the tomato wilt pathogen *Fusarium oxysporum* f.sp. *lycopersici* was the first one to diverge from the last common ancestor, whereas the most recent split was the divergence of *V. dahliae* strains JR2 and VdLs17. The first duplication period that occurred before the divergence of *Colletotrichum higginsianum* affected both the core genome and LS compartments (Fig. 2C). The second duplication event in *V. dahliae* strain JR2 that specifically concerned genes located at LS regions occurred much more recently, after the speciation of *Verticillium alfalfae*. Genes in the LS regions have previously been shown to be particularly relevant for pathogen virulence in *V. dahliae* (de Jonge et al. 2013), suggesting that recent gene duplications are contributing to the evolution of virulence.

**Figure 3.**
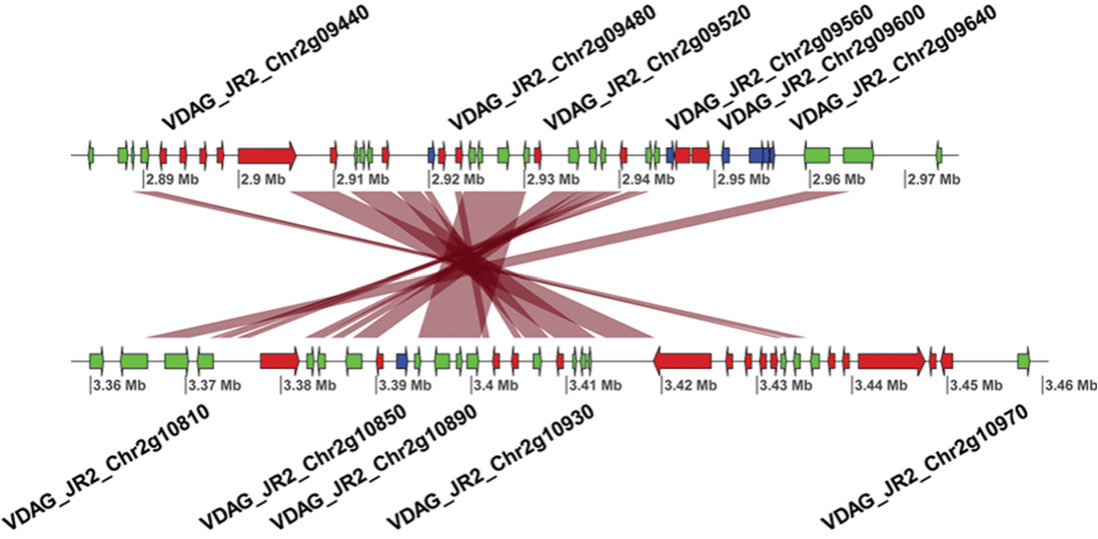
Example of gene losses after segmental duplications within the *V. dahliae* strain JR2 genome. Example of a segmental duplication between LS regions located on Chromosome 2. Red ribbons indicate regions of homology between the two loci. Blue arrows indicate gene models present only at one of the two loci, whereas green and red arrows indicate common genes and transposable elements, respectively.

The recent duplications that affected LS regions have generated raw genetic material that can be subjected to subsequent rapid evolutionary diversification, leading to novel or altered gene functionality, but can also be subject to differential loss of one of the duplicated gene copies (Fig. 3; Supplemental Fig. S9). In general, LS regions in *V. dahliae* strain JR2 display considerable gene loss, because for about 100 of the approximately 400 genes located in LS regions, no ortholog could be detected in *V. dahliae* strain VdLs17. Thus, differential gene loss significantly contributed to the diversification of LS regions. To determine if LS regions display signs of increased gene diversification and selection pressure acting on protein-coding genes, we used the rate of nonsynonymous substitutions per nonsynonymous site (*K*_a_) as well as the ratio of *K*_a_ to *K*_s_ values calculated between orthologous gene pairs of *V. dahliae* strain JR2 and the closest related *Verticillium* species, *V. alfalfae*, as a proxy. Genes located at LS regions display moderately increased *K*_a_ values, as well as *K*_a_/*K*_s_ values, when compared to genes residing in the core genome (median of 0.07 compared to 0.03, and 0.37 compared to 0.18, for LS and core genes, respectively). Although these indicate accelerated sequence divergence of genes located within LS regions, the moderate differences also corroborate previous results (de Jonge et al. 2013; Seidl et al. 2015), suggesting that sequence divergence only plays a minor role in *V. dahliae* genome evolution.

### The *Ave1* effector gene is located in a highly dynamic genomic region

As shown above, highly dynamic LS regions are characterized by frequent gene duplications and differential gene loss. Moreover, effector genes located in LS regions play decisive roles in pathogen-host interactions (de Jonge et al. 2013). For example, Ave1 is an important LS effector that determines *V. dahliae* virulence on various host plants (de Jonge et al. 2012). As expected in a coevolutionary “arms race” (Thomma et al. 2011; Cook et al. 2015), host recognition evolved as host plants that carry the Ve1 immune receptor recognize this effector (Fradin et al. 2009; de Jonge et al. 2012). Whereas race 1 strains of *V. dahliae* are contained by the Ve1 immune receptor, race 2 strains evade recognition due to loss of *Ave1* and are able to infect *Ve1* host plants.

**Figure 4.**
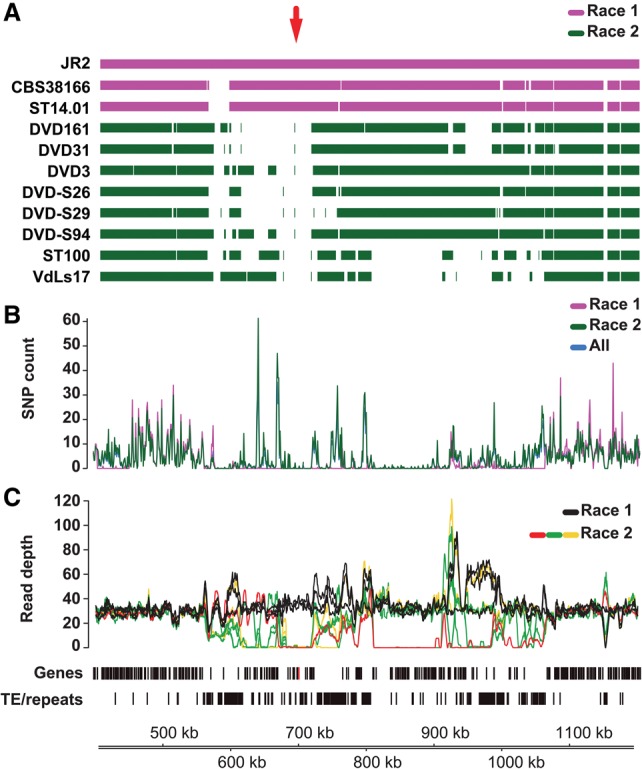
Details of the *Ave1* locus in *V. dahliae* strain JR2. (*A*) Genome assemblies of race 1 and race 2 *V. dahliae* strains were aligned to the reference genome assembly of *V. dahliae* strain JR2. The red arrow indicates the location of the *Ave1* gene. (*B*) Single nucleotide polymorphism (SNP) density (mean number of SNPs per 1 kb) over the *Ave1* locus indicates depletion of SNPs in the *Ave1* region when compared with neighboring regions. (*C*) A large genomic region on Chromosome 5 of *V. dahliae* strain JR2 containing the *Ave1* gene is characterized by presence/absence polymorphisms between strains. Lines indicate the corrected average read depth (per 5-kb window, 500-bp slide) of paired-end reads derived from genomic sequencing of 11 *V. dahliae* strains. Different colors indicate distinct patterns of coverage across the *Ave1* locus. Genes (*Ave1* is marked in red) and transposable elements/repeats (excluding simple repeats) are indicated.

In *V. dahliae* strain JR2, *Ave1* is embedded in a gene-sparse and repeat-rich LS region on Chromosome 5 (approximately 550,000–1,050,000) (Fig. 4). Notably, the average number of single nucleotide polymorphisms (SNPs) inferred from three race 1 and eight race 2 strains of *V. dahliae* is significantly reduced in the area surrounding the *Ave1* locus (between 680,000 and 720,000) when compared with the surrounding genomic regions (Fig. 4B), but also compared to genome-wide SNP levels in LS and core regions (Supplemental Fig. S10), which may be explained by the relatively recent acquisition of this region through horizontal transfer (de Jonge et al. 2012).

*V. dahliae* race 1 strains do not occur as a monophyletic clade in the *V. dahliae* population (Supplemental Fig. S3A; de Jonge et al. 2013). To assess if *Ave1* was gained or lost multiple times, we studied the genomic region surrounding the *Ave1* locus in the LS region. By mapping paired-end reads derived from genomic sequencing of various *V. dahliae* strains onto the genome assembly of *V. dahliae* strain JR2, we observed clear differences in coverage levels between *V. dahliae* race 1 and race 2 strains that carry or lack the *Ave1* gene, respectively (Fig. 4C; Supplemental Fig. S11). Although *V. dahliae* race 1 strains, including JR2, displayed an even level of read coverage over the *Ave1* locus, no read coverage over the *Ave1* gene was found in race 2 strains. Intriguingly, read coverage surrounding the *Ave1* gene revealed that race 2 strains can be divided into three groups, depending on the exact location of the read coverage drop (Fig. 4C; Supplemental Fig. S11B). Whereas one group of isolates does not display any read coverage over a region of ∼40 kb flanking the *Ave1* gene (Supplemental Fig. S11B, colored lines from ∼680 to ∼720 kb), two groups display distinct regions in which the read coverage around the *Ave1* locus drops (Supplemental Fig. S11B, red lines around 668 kb and green lines around 672 kb). In conclusion, the *Ave1* locus is situated in a highly dynamic and repeat-rich region (Fig. 4C), and the most parsimonious evolutionary scenario is that the *Ave1* locus was horizontally acquired once, followed by multiple losses in independent lineages that encountered host plants that carried Ve1 or functional homologs of this immune receptor (Thomma et al. 2011; de Jonge et al. 2012; Zhang et al. 2014; Song et al. 2016).

### Lineage-specific genomic regions in *Verticillium dahliae* contain active transposable elements

Although the activity of TEs is not associated with the formation of extensive genome rearrangements, LS regions are highly enriched for TEs, and their presence and potential activity may contribute to accelerated evolution of these genomic regions. In *V. dahliae*, the most abundant class of TEs are retrotransposons that transpose within the genome using an RNA intermediate (Supplemental Table S1; Wicker et al. 2007; Faino et al. 2015). We assessed TE dynamics by querying the transcriptional activity of TEs using in vitro RNA-seq data derived from *V. dahliae* strain JR2 (de Jonge et al. 2012). Notably, the majority of TEs in *V. dahliae* are not transcribed and thus likely not active (Supplemental Table S1), whereas transcribed and therefore likely active TEs are found in LS regions—171 of 280 TEs in LS regions (61%) are expressed (log_10_[RPKM +1] >0) (Supplemental Fig. S12).

**Figure 5.**
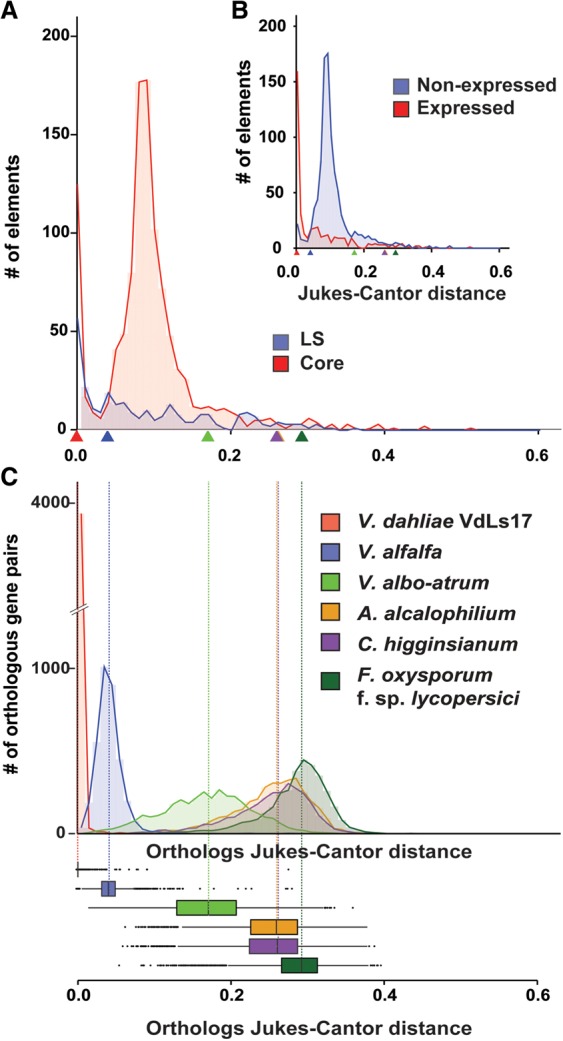
Dynamics of transposable elements in the genome of *Verticillium dahliae* strain JR2*.* (*A*) The divergence time of transposable elements identified in the genome of *V. dahliae* strain JR2 (Faino et al. 2015) was estimated using the Jukes-Cantor distance calculated between repeat copies and their consensus sequence. The distributions of divergence times between transposable elements located in the core genome (red) and in the LS regions (blue) differ. Estimations of speciation events in the evolutionary history of *V. dahliae* are indicated by triangles based on analyses in *C*. (*B*) The distributions of divergence times between expressed/active (log_10_[RPKM+1] >0) transposable elements (red) and nonexpressed (blue) transposable elements differ. Estimations of speciation events are indicated by triangles. (*C*) Speciation events are estimated by calculating the Jukes-Cantor distance for orthologous gene pairs based on genes from *V. dahliae* strains JR2 and their respective orthologs in the other genomes. Distributions and median divergence times between 1:1:1 orthologous pairs, displayed by box plots, were used to estimate relative speciation events.

To further assess if and how TEs influence the evolution of LS regions, we explored TE dynamics in the genome of *V. dahliae* strain JR2. Each copy of a TE in the genome is derived from an active ancestor that, once transposed and integrated into the genome, accumulates mutations that over evolutionary time will render the TE inactive. The relative age of individual TEs can thus be estimated based on sequence divergence from a consensus sequence that can be derived from present-day copies of any given TE. Using the Jukes-Cantor distance (Jukes and Cantor 1969), which corrects the divergence between TEs and their consensus sequence for multiple substitutions, we estimated the divergence times for TEs in the *V. dahliae* strain JR2 genome (Fig. 5). This analysis showed that TEs primarily transposed and expanded in two distinct periods (Fig. 5A). Notably, a considerable amount of “younger” TEs, i.e., with small Jukes-Cantor distance (Jukes and Cantor 1969) to their consensus sequence, localizes in LS regions, whereas the majority of “older” TEs resides in the core genome (Fig. 5A). In line with this observation, 90 of 280 TEs in LS regions (i.e., 32%) display <0.025 nucleotide divergence from the consensus sequence, whereas 151 of 1101 TEs in the core (i.e., 14%) display such low levels of divergence. Similarly, genome-wide, 202 of the 390 transcriptionally active TEs (i.e., 52%) display <0.025 divergence, whereas only 39 of 991 transcriptionally silent TEs (i.e., 4%) display such low levels of divergence (Fig. 5B). Additionally, the relative age of individual TEs belonging to the LTR class was estimated based on sequence divergence between the distal LTR sequence motifs using the Jukes-Cantor distance (Fiston-Lavier et al. 2012). Despite the relatively low amount of complete LTR-type TEs found in the genome of *V. dahliae* strain JR2, this analysis showed a similar age distribution (Supplemental Fig. S13). Next, we attempted to determine the relative period in which the majority of TEs in the genome of *V. dahliae* strain JR2 transposed. To this end, we derived Jukes-Cantor distributions for orthologous genes of *V. dahliae* strain JR2 and individual closely related fungi as a proxy of divergence between these species (Fig. 5C). These distributions display the same pattern of species divergence when derived from phylogenetic analyses (Supplemental Fig. S14) as well as from the *K*_s_ distributions between orthologous gene pairs (Fig. 2C). By comparing the Jukes-Cantor distributions derived from TEs and from orthologous genes, we revealed that older TEs transposed before the separation of *V. dahliae* and *V. alfalfa*, whereas the younger TEs transposed after *V. dahliae* speciation (Fig. 5C). Notably, younger TEs tend to be transcriptionally active, and older, more diverged TEs tend to be transcriptionally silent (Fig. 5B). Thus, the expansion of younger TEs is recent and primarily concerns the active TEs localized at LS regions (Fig. 5), strongly suggesting that TE transpositions contribute to the genetic plasticity of LS regions.

## Discussion

It was previously shown that genetic rearrangements occur in fast-evolving LS regions of the genome that are enriched in TEs (Klosterman et al. 2011; de Jonge et al. 2013). We now show that TEs are enriched near rearrangement breakpoints and play a role in homology-based recombinations. We furthermore show that recent genetic duplications are enhanced in LS regions, against the background of an older episode of duplication events for the “core” genome. Finally, we show that LS regions display signs of TE activity that may contribute to plasticity of those regions.

Many plant pathogens contain a so-called “two-speed” genome, where effector genes reside in genomic compartments that are considerably more plastic than the core genome, facilitating the swift evolution of effector catalogs that are required to be competitive in the host-pathogen arms race (Raffaele and Kamoun 2012; Dong et al. 2015). Generally, effector compartments are enriched in transposable elements (TEs), and it has been speculated that they promote genomic flexibility and drive accelerated evolution of these genomic compartments (Gijzen 2009; Haas et al. 2009; Ma et al. 2010; Raffaele et al. 2010; Rouxel et al. 2011; Raffaele and Kamoun 2012; de Jonge et al. 2013; Wicker et al. 2013; Grandaubert et al. 2014; Seidl and Thomma 2014; van Hooff et al. 2014; Dong et al. 2015; Faino et al. 2015; Seidl et al. 2015). This phenomenon is not confined to plant pathogens, as the genome of the ant *Cardiocondyla obscurior* similarly contains large TE islands enriched for genes with roles in adaptation to novel habitats that display elevated levels of single-nucleotide polymorphisms and structural variations (Schrader et al. 2014). In plants and animals, TEs are considered significant drivers of genome architecture (Bailey et al. 2003; Feschotte and Pritham 2007; Cordaux and Batzer 2009; Wicker et al. 2010; Lisch 2013). Nevertheless, the exact role of TEs in genome evolution of filamentous pathogens remained unknown (Raffaele and Kamoun 2012; Dong et al. 2015).

Here, we identified ∼2-Mb repeat-rich, lineage-specific (LS) regions between two *V. dahliae* strains that are significantly enriched in TEs and contain all thus far functionally analyzed effector genes, including *Ave1*. Moreover, we also determined a significant number of synteny breakpoints that are associated with genomic rearrangements to high resolution, most of which we could reconstitute in detail. Although the previously determined association between LS compartments and the occurrence of genomic rearrangements was overestimated due to major errors in the publicly available genome assembly of *V. dahliae* strain VdLs17 (Klosterman et al. 2011), for which we recently revealed a considerable number of erroneous chromosomal inversions (Faino et al. 2015), we still observed that three of four large-scale LS regions are associated with chromosomal rearrangements. We were not able to associate every LS region with a genomic rearrangement, and we were also not able to exactly reconstitute each genomic rearrangement. However, it can be anticipated that complex rearrangements occurred in which genetic material in close proximity was lost. Furthermore, rearrangements may have occurred over longer evolutionary timescales, and subsequent rearrangement events may have erased “scars” of previous rearrangements. Thus, although not every genomic rearrangement can be associated to an LS region, large-scale genomic alterations appear to be the driving force for LS region formation in *V. dahliae*.

Intriguingly, although the majority of TEs in *V. dahliae* is transcriptionally silent and thus inactive, transcriptionally active and thus likely actively transposing TEs were observed in LS regions. This observation was corroborated using a very conservative approach to map RNA-seq reads to limit the effect of reads mapping to multiple locations in the genome. Transcription and specific induction of TE activity has been observed previously in *V. dahliae* strain VdLs17 (Amyotte et al. 2012). These independent observations are further corroborated by our dating analyses, suggesting that TEs that localize in LS regions are significantly younger when compared with those residing in the core genome. We propose that TEs and their activity contribute to genomic diversity by, e.g., inducing large-scale genomic duplications, as observed within the LS regions in *V. dahliae*. In plants, TE insertions into chromosomes induce double-strand breaks, which are subsequently repaired using ectopic DNA as a filler, thereby leading to the duplication of genomic material that can subsequently diverge, often involving gene loss (Wicker et al. 2010). Intriguingly, the LS effector *Ave1* is located in an LS region with several TEs in its direct vicinity (de Jonge et al. 2012), and clear evidence for repeated loss in the *V. dahliae* population was obtained in our study. Similarly, it has been hypothesized that ectopic genomic rearrangements caused by homology-based DNA repair pathways, possibly passively mediated by TEs, drive the frequent loss of the *Avr-Pita* effector gene in isolates of the rice blast fungus *Magnaporthe oryzae* that encountered the *Pita* resistance gene of rice (Orbach et al. 2000; Chuma et al. 2011). Similar processes likely contribute to the frequent recovery of *Avr-Pita* in *M. oryzae* strains relieved from selection pressure of Pita, leading to translocation and duplications of this effector gene in dynamic genomic regions (Chuma et al. 2011).

Genome-wide studies in several fungi aiming to study chromatin revealed that TE-rich regions are generally associated to highly condensed chromatin that restricts transcription and TE activity (Lewis et al. 2009; Connolly et al. 2013; Galazka and Freitag 2014). Therefore, TEs can influence the expression of neighboring genes such as effectors, as they can direct the formation of heterochromatic regions (Lewis et al. 2009). In the saprophytic fungus *Neurospora crassa*, heterochromatin formation at TEs is directed by remnants of Repeat Induced Point mutations (RIP), a premeiotic process that actively induces point mutations in TEs (Selker 1990; Lewis et al. 2009, 2010). In the pathogenic fungus *Leptosphaeria maculans*, effectors are located in TE-rich regions that were subjected to extensive RIP mutations and display signatures of nucleotide mutations caused by the RIP process, fostering rapid effector diversification (Fudal et al. 2009; Daverdin et al. 2012). However, as *Verticillium* spp. are considered asexual, it is not unexpected that effectors in LS regions do not display evidence for RIP mutations. Despite the fact that LS regions do not show overrepresentation of secreted genes, we previously observed that effector genes residing in the LS regions are considerably overrepresented in the *V. dahliae* transcriptome upon plant infection (de Jonge et al. 2013). Potentially, since several TEs in LS regions are transcriptionally active, LS regions may either not yet be targeted by heterochromatin formation or carry different chromatin marks as core genomic compartments. Chromatin-based regulation of effector genes and genes encoding other virulence factors has been observed in several pathogenic fungi (Connolly et al. 2013; Qutob et al. 2013; Chujo and Scott 2014; Soyer et al. 2014, 2015). Thus, further insight into chromatin biology of *V. dahliae* will be instrumental to significantly enhance our knowledge of the evolution of its two-speed genome and on virulence.

## Methods

### Genome annotation and dynamics

Gene predictions for the genome assembly of *V. dahliae* strain JR2 (Faino et al. 2015) was performed using Maker2 software (Holt and Yandell 2011) with multiple independent evidence, including transcriptomic data, to support gene annotation (Klosterman et al. 2011; de Jonge et al. 2012, 2013; Seidl et al. 2015). For details, see Supplemental Methods. Homology between protein-coding genes of 13 fungal species was assessed using OrthoMCL (Li et al. 2003). Orthologous gene pairs between fungal species and paralogous gene pairs within each individual species were extracted from the OrthoMCL gene families. *K*_s_ values between gene pairs, as defined by OrthoMCL families, were calculated using the *K*_a_*K*_s__Calculator 2.0 package (Wang et al. 2010).

Repetitive elements were identified as described in Faino et al. (2015). Expression of repetitive elements was assessed based on RNA sequencing data derived from *V. dahliae* strain JR2 grown in in vitro media (Czapek Dox) (de Jonge et al. 2012). Paired end reads were mapped onto the genome assembly of *V. dahliae* strain JR2 using TopHat2 (Trapnell et al. 2009), summarized, and reported as reads per kilobase of transcript per million mapped reads (RPKM). To estimate divergence time of transposable elements, each individual copy of a transposable element was aligned to the consensus of its family. The sequence divergence between transposable elements and the consensus was corrected using the Jukes-Cantor distance, which corrects the divergence (*p*) by the formula *d* = −3/4log_e_(1−4/3*p*) (Jukes and Cantor 1969). For more details, see Supplemental Methods.

### Identification of genomic rearrangements

The genome assemblies of *V. dahliae* strain JR2 and VdLS17 are available from NCBI under the assembly number GCA_000400815.2 and GCA_000952015.1, respectively (Faino et al. 2015). Whole-genome alignments between chromosomes of the genome assemblies of *V. dahliae* strains JR2 and VdLS17 were performed using MUMmer (Kurtz et al. 2004) and mined for genomic rearrangements and associated synteny breakpoints in *V. dahliae* strain JR2. Synteny breakpoints were further refined by mapping PacBio long-sequencing reads derived by genomic sequencing of *V. dahliae* strain VdLS17 to the genome of *V. dahliae* strain JR2 using BLASR (Chaisson and Tesler 2012), followed by manual refinement. To assess the presence or absence of genomic rearrangements in other *V. dahliae* strains, paired-end reads derived from genome sequencing (PRJNA169154) (de Jonge et al. 2012) were mapped onto the genome of *V. dahliae* strain JR2 using BWA (Li and Durbin 2010), and genomic regions surrounding the identified genomic rearrangements (±4 kb) were visually evaluated for the quantity of concordantly and discordantly mapped reads as well as orphan reads. For more details, see Supplemental Methods.

### Identification and analyses of highly dynamic genomic regions

Whole-genome alignments between chromosomes of the complete genome assemblies of *V. dahliae* strains JR2 and VdLS17 (Faino et al. 2015) were performed using MUMmer (Kurtz et al. 2004). LS regions were manually defined by identifying regions accumulating alignment breaks and TEs. The presence/absence analysis of the *Ave1* locus was performed by aligning paired-end reads from DNA sequencing of 11 *V. dahliae* strains (PRJNA169154, including JR2) (de Jonge et al. 2012) to the assembled genome of *V. dahliae* strain JR2 using BWA (Li and Durbin 2010). Raw read depth per genomic position was averaged per genomic window—window-size 5 kb, slide 500 bp (Fig. 4C), and window-size 500 bp, slide 100 bp (Supplemental Fig. S11), respectively—and subsequently G+C corrected as previously described (Yoon et al. 2009). Genomic reads of each individual additional *V. dahliae* strain were assembled using A5 pipeline (Tritt et al. 2012), and genome assemblies were subsequently aligned to the genome assembly of *V. dahliae* strain JR2 genome using MUMmer (Kurtz et al. 2004). For more details, see Supplemental Methods.

Single nucleotide polymorphisms (SNPs) were identified using GATK v2.8.1 (DePristo et al. 2011). SNPs derived from different strains were summarized in nonoverlapping windows of 1 kb, and the number of SNPs derived from individual strains was averaged per window. Absence of a SNP in a particular strain was only considered if the corresponding position displayed read coverage. For more details, see Supplemental Methods.

## Competing interest statement

A.H.J.W. is a full-time employee of KeyGene N.V., a company offering next-generation sequencing services, including PacBio sequencing.
